# Examining the Intersectional and Structural Issues of Routine Healthcare Utilization and Access Inequities for LGB People with Chronic Diseases

**DOI:** 10.3390/ijerph22121830

**Published:** 2025-12-06

**Authors:** Shiya Cao, Mehreen Mirza, Sophia Silovsky, Nicole Tresvalles, Lucia Qin, Sarah Susnea

**Affiliations:** Statistical and Data Sciences, Smith College, 10 Elm Street, Northampton, MA 01063, USAsophiasils29@gmail.com (S.S.);

**Keywords:** quantitative intersectional analysis, sexual orientation, discrimination, healthcare utilization and access

## Abstract

In the United States, although the gaps in health insurance coverage by sexual orientation have been closing since the implementation of the Affordable Care Act and legalization of same-sex marriage, the LGB group (i.e., lesbian, gay, bisexual) continues to report healthcare utilization and access inequities such as more delayed or unmet care. The extant research has often examined healthcare utilization and access inequities due to affordability (e.g., out-of-pocket costs). However, healthcare utilization and access inequities are only partially explained by cost reasons; there are non-cost reasons that have not been adequately empirically examined. The present study innovatively includes discrimination structural variables to understand how social structure is associated with healthcare utilization and access inequities of LGB people. It focuses on two routine health services—regular check-ups and prescription medications—for LGB people who have chronic diseases. Additionally, sexual orientation may intersect with sex assigned at birth (sex, hereafter, i.e., male, female) to impact healthcare utilization and access inequities. The current study applies quantitative intersectional analysis to understand healthcare utilization and access inequities from a sexual orientation and sex intersectional lens and for easier and clearer interpretations of intersectional results and more actionable policy implications for inter-categorical groups. Using the 2023 National Health Interview Survey (weighted *N* = 136,231,053), we conducted quantitative intersectional analysis for logistic regression using complex survey data. First, we fit a series of logistic regression models with sexual orientation–sex interactions for routine healthcare utilization and access outcomes, adjusting for covariates. Second, we calculated average marginal predictions for inter-categorical groups by interacting sexual orientation and sex and other covariates. Third, we computed risk ratios of average marginal predictions for all the covariates. Lastly, we examined the interaction of inter-categorical groups/sexual orientation and structural variables. Our results show that experiencing a higher level of discrimination is positively associated with underutilization of regular check-ups and lower access to prescription medications, and this effect is stronger for LGB people. Further, LGB women are least likely to utilize regular check-ups and LGB men are least likely to access prescription medications among the inter-categorical groups. Highlighting structural issues of healthcare utilization and access offers new evidence on healthcare utilization and access inequities that can inform policies for raising awareness of and addressing structural issues. The intersectional analyses suggest that relevant policies target LGB women and LGB men.

## 1. Introduction

In the United States (U.S.), although the gaps in health insurance coverage by sexual orientation have been closing since the implementation of the Affordable Care Act (ACA) and legalization of same-sex marriage, the LGB group (i.e., lesbian, gay, bisexual) continues to report healthcare utilization and access inequities such as more delayed or unmet care [1,2]. The extant research has often examined healthcare utilization and access inequities due to affordability (e.g., out-of-pocket costs) [3,4,5]. However, healthcare utilization and access inequities are only partially explained by cost reasons [4]; there are non-cost reasons that have not been adequately empirically examined [3,4]. The present research answers the need to build upon the literature in understanding social structures attributed to healthcare utilization and access inequities of LGB people.

Discrimination, a social structure issue, has received increasing attention in the literature as a potential contributor to lower utilization of and access to health services [6,7]. The LGB group is a vulnerable population who may experience more discrimination because of their sexual orientation within and outside the healthcare system [1]. While a few existing studies examined the association between discrimination experienced within the healthcare system and healthcare utilization/access, a smaller body of research explored the association between perceived everyday discrimination outside the healthcare system and healthcare utilization/access [7]. Everyday discrimination is defined as “relatively minor, but chronic and fairly common, day-to-day experience of slights and insults” (p. 1045) [7]. A review paper on health and healthcare of sexual and gender minorities suggested that people who face discrimination may delay their care and show up in medical spaces potentially more ill and face more discrimination [1]. Using a cross-sectional sample, Fazeli Dehkordy et al. evaluated the association between everyday discrimination and health service use for women in the U.S. [7]. They discovered that experiencing everyday discrimination was associated with higher odds of more frequent healthcare visits [7]. There is a possibility that encounters with healthcare is a source of discrimination [7]. However, due to the cross-sectional design, the authors were inconclusive in terms of the direction of the causal association between everyday discrimination and health service use and suggested that future studies elucidate this further [7]. Another paper on LGB veterans’ use of Veterans Healthcare Administration (VHA) services found mixed results of the relationship between social structure and healthcare utilization: while LGB veterans who experienced at least one interpersonal trauma during the military due to their sexual orientation were statistically significantly more likely to have utilized VHA in the past year or ever, LGB veterans who had at least one stressful experience initiated by the military to investigate or punish their LGB status were statistically significantly less likely to have utilized VHA in the past year, but this factor was not statistically significantly associated with lifetime VHA utilization [8]. Analyzing a sample of 149 LGB people who have chronic diseases, Brooks et al. found that a higher level of anticipated discrimination was associated with increased depressive symptoms and, in turn, poorer treatment adherence (e.g., not taking medications prescribed by a healthcare provider) [9]. Stressors such as depressive symptoms may lead to disengaged coping behaviors such as avoiding the healthcare system [9]. Similarly, Casagrande et al. posited that internalization of discrimination may increase psychosocial or attitudinal stressors (e.g., depression) that may be associated with underutilization of health services and assessed this relationship for African American and White people in a racially integrated community [6]. They found that discrimination experiences were associated with lower levels of healthcare utilization, regardless of being African American or White [6]. This may confirm and extend the authors’ assumption that if discrimination acts as a dominant stressor, then it may have similar impact on one’s behaviors regardless of their race [6]. The authors also alluded that in most of their models the effects were greater for White people, and it may be because experiencing everyday discrimination is less normative among White people; when they do experience it, the association between discrimination and health services utilization is stronger [6]. This gives us implications that it is important to study the interaction of discrimination and social identities, for instance, whether experiencing discrimination has a stronger effect on LGB people than straight people with respect to not utilizing/accessing healthcare, so we can have a more nuanced understanding of the role of social structure in healthcare utilization/access. Building upon the extant literature, the current research empirically examines if experiencing discrimination would be associated with lower levels of healthcare utilization and access for LGB people using a nationally representative sample of the U.S. adult population.

In addition, sexual orientation may intersect with sex assigned at birth (sex, hereafter, i.e., male, female) to impact healthcare utilization and access inequities [2]. For example, HIV/AIDS care services targeted toward LGB men (also known as gay and bisexual men) may increase their healthcare utilization [2]. Additionally, masculine gender role norms perpetuating the idea that men are supposed to be tough and not seek help may contribute to straight men having lower rates of healthcare utilization compared to LGB men [2,10]. However, some research also reports that bisexual men are less likely to afford prescription medications and medical testing than straight men [3]. On the other hand, LGB women (also known as lesbian and bisexual women) do not have targeted healthcare programs and may be more directly driven by minority stress and social structure factors, resulting in lower rates of healthcare utilization compared to straight women [2,3]. Since overall LGB people (also known as LGB women and LGB men) are less likely to utilize and access healthcare compared to straight people [2,3,5,11], we hypothesize that LGB women are least likely to utilize and access healthcare among the inter-categorical groups by interacting sexual orientation and sex.

The current study focuses on people who have chronic diseases. Chronic diseases are defined as diseases that last at least one year and require ongoing medical attention or limit activities of daily living, or both [12]. People who have chronic diseases often require routine check-ups (e.g., blood pressure, blood sugar checks) with their doctors to manage chronic conditions [13,14,15]. Research shows that the increasing number of visits with a primary care provider is associated with better care experiences for people who have chronic diseases [16]. Further, it is essential for people who have chronic diseases to take prescription medications at the right time [13,17]. Studies indicate that LGB people are more likely to have chronic diseases (e.g., chronic heart diseases, depression, stroke, hypertension) as compared to straight people [3,5,18,19]. However, LGB people are less likely to utilize and access routine healthcare [1,2,3]. In particular, LGB women are less likely to utilize routine physical exams, and bisexual men are less likely to afford prescription medications than their straight counterparts [2,3]. As a critical first step, the present research examines the relationship between the routine healthcare utilization and access (e.g., regular check-ups and prescription medications) of people who have chronic diseases, perceived discrimination, and the intersection of sexual orientation and sex.

### 1.1. Discrimination and Healthcare Utilization and Access

Everyday Discrimination and Anticipated Discrimination as structural variables have scarcely been explored in the context of healthcare utilization and access. Day-to-day experience of slights and insults and heightened vigilance for those slights and insults may be associated with lower utilization of and access to health services because of psychosocial or attitudinal stressors (e.g., depression) [6]. LGB people who experience discrimination and disrespect at workplaces, stores, hospitals, or elsewhere may be worried about seeking services at social institutions including healthcare institutions [20]. Related to discrimination, the theory of stigma, as people who are stigmatized often experience discrimination, suggests that fear of being stigmatized or devalued is a barrier to utilizing healthcare services for LGB people [21,22,23]. Currently, heteronormative beliefs solely based on the sexual orientation of an individual still exist among healthcare providers [14,23]. This may cause increasing worries and distrust for LGB people, resulting in their delays or avoidance of healthcare utilization. On the other hand, everyday discrimination or anticipated discrimination from employment, income, education, health insurance, and so on disadvantage LGB people [20,24]. They often have fewer of those resources as compared to their straight peers [20,24] and may need to make decisions on where to spend their limited resources. Due to this, LGB people may delay or bypass access to healthcare (e.g., prescription medications). Understanding those discrimination structural issues and how perceived discrimination may be associated with healthcare utilization/access is important for addressing healthcare utilization and access inequities that still exist between LGB people and their straight peers. It would help inform interventions that increase awareness of and address LGB people’s experiences of discrimination both within and outside the healthcare system.

### 1.2. Healthcare Utilization and Access by Sexual Orientation and Sex and Quantitative Intersectional Analysis

The present study aims to understand healthcare utilization and access inequities across inter-categorical groups by interacting sexual orientation and sex. Intersectionality, originally coined by Kimberlé Crenshaw [25], refers to how multiple systems of oppression interact in shaping individual experiences. The intersectional approach examines heterogeneity across inter-categorical groups and expands cumulative disadvantages [26]. Quantitative intersectionality analysis has been increasingly incorporated into the epidemiology and public health field [27,28,29,30]. We define quantitative intersectional analysis as a theoretical framework for how to think about data analysis in a way that posits that experiences at an intersection must be considered jointly and understands the relevance of different statistical methods to intersectional applications and interpretations. Studying healthcare utilization and access inequities for LGB people using a quantitative intersectional approach is an important first step to understand the within-group differences in the health inequities diverse underrepresented populations face and provide more actionable policy implications for inter-categorical groups.

This study focuses on quantitative intersectional methods for logistic regression because the outcome variables are dichotomous. For logistic regression or other log-scale regression with product terms, we need to implement several steps for easier and clearer interpretations of intersectional results [27,31]. Extant studies have often not systematically and comprehensively applied quantitative intersectional methods for logistic regression in empirical studies to show their helpfulness. In the present study, we apply these methods to examine healthcare utilization and access inequities across inter-categorical groups, including sexual orientation and sex.

Suppose a sample of size *n* is obtained from a population-based sample survey. Let $y_{j}$ (*j* = 1, …, *n*) be the binary outcome variable of interest. The logistic regression model with *R* groups, *n* observations, p-dimensional vectors of covariates $x_{j},$ and regression coefficients *α* and *β* is as follows (Equation (1)):

(1) $$log\frac{P(y_{j}=1)}{1-P(y_{j}=1)}=\;\alpha_{i}I_{ij}+\beta x_{j}$$

where *i* = 1, …, *R*, $I_{rj}=1$ if the *j*th observation in group *r* and 0 is otherwise.

After fitting logistic regression models, we first calculate average marginal predictions for groups defined by sexual orientation and sex and other covariates. The average marginal prediction for group *r* is the average predicted response if all the observations were in group *r* [32] (Equation (2)).

(2) $$M_{r}=\frac{1}{n}\sum_{j=1}^{n}\left(\frac{exp({\hat{\alpha }}_{r}+\hat{\beta }x_{j})}{1+exp({\hat{\alpha }}_{r}+\hat{\beta }x_{j})}\right)$$

For complex survey data, we can easily account for weights (Equation (3)) in the previous Equation (2).

(3) $$M_{r}=\frac{\sum_{j=1}^{n}w_{j}\left(\frac{exp({\hat{\alpha }}_{r}+\hat{\beta }x_{j})}{1+exp({\hat{\alpha }}_{r}+\hat{\beta }x_{j})}\right)}{\sum_{j=1}^{n}w_{j}}$$

Using average marginal predictions may have several advantages: (1) The average marginal predictions may convey the scale of group differences better than regression coefficients [32]. Comparisons of predicted outcomes between groups can be easily made using average marginal predictions, after adjusting for differences in covariate distributions between the groups [33]. (2) In some applications, there is interest in the magnitude of the effects of inclusion of certain covariates in the model on group differences. By performing the analysis with and without the covariate, one can determine the changes in the average marginal prediction for each of the groups [32]. (3) This method is based on published and well-accepted procedures [32,33]. (4) They can easily be produced using svypredmeans from the survey package using the R programming software (R version 4.5.2, open-source software, https://www.r-project.org/), accounting for the complex survey design (weighting, clustering, and stratification).

After calculating average marginal predictions, we need to compute risk ratios of average marginal predictions. Risk ratio indicates how many times higher the risk of the outcome was in people who were exposed to the risk factor. Risk measures the proportion of people who developed the outcome. On the other hand, odds ratio indicates how many times higher the odds of the outcome was in people who were exposed to the risk factor. Odds compare the number of people who developed the outcome to the number who did not. It is risk ratio that people more intuitively understand as a measure of association. Additionally, when an outcome is common (e.g., not using regular check-ups within the last 2 years in Table 1), the odds ratio will exaggerate the risk ratio. It is recommended that, for quantitative intersectional analysis for logistic regression, researchers familiarize themselves with and use methods that compute risk ratios with cross-sectional data (e.g., the National Health Interview Survey (NHIS) we are using in this study) [27].

### 1.3. Hypotheses

Inter-categorical groups.

**Hypothesis 1.** 
*LGB women are least likely to utilize/access routine healthcare among the inter-categorical groups by interacting sexual orientation and sex*.

Structural variables.

**Hypothesis 2.** 
*People who report experiencing higher levels of discrimination are less likely to utilize/access routine healthcare than counterparts who report experiencing lower levels of discrimination*.

Sexual orientation × Structural variables.

**Hypothesis 3.** 
*Experiencing higher levels of discrimination will have a stronger effect on LGB people than their straight peers with respect to not utilizing/accessing routine healthcare because LGB people are in a less privileged sexual orientation and social position, and discrimination may lead to more psychosocial stressors for them, thereby associating with their lower utilization of and access to health services [2,6,34]*.

## 2. Materials and Methods

### 2.1. Data Source

The analysis used data from the adult sample of the 2023 NHIS, a national household survey conducted by the National Center for Health Statistics, Center for Disease Control and Prevention. This survey was conducted as a face-to-face interview, on the U.S. civilian noninstitutionalized population, using geographically clustered sampling techniques [35]. The data collection was a continuous process, from January to December, and information is compiled from the survey answers. Interviews were typically conducted in respondents’ homes, but follow-ups to complete interviews may be conducted over the telephone [35]. The NHIS’s main purpose is to monitor and track the health status and healthcare of the U.S. population [35]. It has been used to capture health and other demographic characteristics, health behaviors, health insurance status, and healthcare utilization outcomes [1,5,33,36]. In the 2023 survey, five items for Everyday Discrimination and four items for Anticipated Discrimination were added to the adult sample of the NHIS to better understand how discrimination relates to health outcomes [37]. Since 2013, the NHIS has included a sexual orientation question in its adult sample, allowing scholars to examine healthcare utilization and access inequities by sexual orientation. For the year 2023, 29,522 adults aged 18 years or older were sampled. Since the NHIS data is cross-sectional data that is collected from a population at a single point in time, our study as a cross-sectional quantitative analysis can understand intersectional and structural issues of healthcare utilization and access for LGB people but should be cautious about making causal inference.

### 2.2. Sample Description

We included adults aged 26 to 64, dropping the sample to 17,565. The choice of age range (26–64) intends to focus on working-age people who have their own health insurance. The ACA requires adults to have health insurance starting at age 26, and adults who are under 26 may be on their parents’ health insurance plans. On the other hand, people who are over 64 may be retired and have different health insurance plans that would complicate our analysis.

We further restricted our sample to people who have chronic diseases, dropping the sample to 15,250. The 2023 NHIS asked a series of questions related to health conditions (most are related to chronic health conditions); for example, “have you ever been told by a doctor or other health professional that you had Chronic Obstructive Pulmonary Disease (COPD), Emphysema, or Chronic Bronchitis?” We examined those questions and selected all the chronic health conditions according to the Center for Disease Control and Prevention [38] except for chronic vision conditions (e.g., glaucoma). The reasons why are as follows: first, many of the vision conditions are often related to aging and that is out of the age range for the present research; second, we do not specifically focus on vision care in this research and will present that for future research. Additionally, we included Hepatitis because infectious diseases may become chronic and LGB people may be at heightened risk for infectious disease transmission due to systemic discrimination. We also included Immunosuppression because this health condition may be related to both chronic and infectious diseases. Further, we included people who reported serious mental health conditions (i.e., feel anxious/depressed daily, take prescription medication for anxiety/depression, or feel very anxious/depressed when thinking about the last time they felt anxious/depressed) because these conditions may be chronic even if mental disorders were not diagnosed and can involve or exacerbate physical conditions for which people would seek care. Thus, the following physical, mental, and infectious disease-related chronic diseases were included in our sample:

Physical: Asthma; COPD, Emphysema, or Chronic Bronchitis; Hypertension; Coronary Heart Disease; Prediabetes; Gestational Diabetes; Diabetes; Arthritis; Cancer; Chronic Pain; Dementia; Epilepsy; Chronic Fatigue Syndrome; Stroke; Crohn’s Disease; Psoriasis; Ulcerative Colitis; Immunosuppression.

Mental: Anxiety Disorder; Depression; Serious Mental Health Conditions.

Infectious diseases: Long COVID; Hepatitis.

Furthermore, we excluded responses that missed any of the discrimination items, further dropping the sample to 14,694 (weighted *N* = 136,231,053). The 2023 NHIS included two discrimination variables: Everyday Discrimination and Anticipated Discrimination. For Everyday Discrimination, it included five items from the Everyday Discrimination Scale to ask how often adults experience unfair treatment [37]. Participants were asked, “in your day-to-day life how often have any of the following things happened to you: (1) You are treated with less courtesy or respect than other people; (2) Compared to other people, you receive poorer service at restaurants or stores; (3) People act as if they think you are not smart; (4) People act as if they are afraid of you; and (5) You are threatened or harassed”. For Anticipated Discrimination, it included four items from the Heightened Vigilance Scale to ask how often adults anticipate and prepare for discrimination [37]. Participants were asked “in your day-to-day life how often do you (1) try to prepare for possible insults from other people before leaving home; (2) feel that you always have to be very careful about your appearance to receive good service or avoid being harassed; (3) carefully watch what you say and how you say it; and (4) try to avoid certain social situations and places (These could be situations like social gatherings but they could also be places like stores, banks, or hospitals or even governmental offices like the Department of Motor Vehicles)”. Only 3.6% of the responses missed one or more than one discrimination item among the nine items from everyday discrimination and anticipated discrimination. We excluded those responses because when we calculated the mean scores of Everyday Discrimination and Anticipated Discrimination, respectively (explained later in Section 2.3.2), missing data would skew the mean scores.

When investigating access to prescription medications, we filtered the sample to people who took prescription medications within the past 12 months (see details in Section 2.3.1), resulting in *N* = 10,694 (weighted *N* = 97,786,893). As shown in Table 2, the percentage distributions of the variables we examined were overall similar between the sample for regular check-ups and the sample for prescription medications.

### 2.3. Breakdown of Variables

The present study examined the associations between sexual orientation, sex, discrimination, and routine healthcare utilization/access. It focused on the intersectional and structural issues of healthcare utilization/access, so sexual orientation, sex, and discrimination structural variables were included in the model. Other covariates including age, ethnicity, marital status, education, poverty status, urban–rural classification, insurance coverage source, deductible, difficulty paying medical bills, having a usual place for care, transportation barrier, and self-rated health were chosen based on existing papers [3,11,39,40,41]. For all the variables given below, we recoded “Not Ascertained”, “Refused”, and “Don’t Know” as “NA” to simplify non-answered questions.

#### 2.3.1. Outcome Variables

The first outcome variable was whether or not a person had a wellness visit, physical, or general-purpose check-up within the last 2 years. It is recommended that people under age 45 routinely check up with their doctors every one to three years and once a year for those who are 45 or older [42]. Given the age range (26–64) of the present study, we chose the 2-year timeframe. We created this outcome variable by utilizing the two variables “Time since last saw doctor” and “Was last visit a wellness visit, physical, or general-purpose check-up” in the 2023 NHIS. People who reported to have had a wellness visit, physical, or general-purpose check-up within the last 2 years were selected as our reference group, coded as 0; people who reported to not have had a wellness visit, physical, or general-purpose check-up within the last 2 years were coded as 1.

The second outcome variable was whether or not a person had full prescription medications without delay within the past 12 months. The 2023 NHIS does not include a variable related to respondents who were not prescribed any medications. Since 73% of our sample of people who have chronic diseases took prescription medications within the past 12 months, as mentioned in Section 2.2, when investigating access to prescription medications, we further filtered the sample to people who took prescription medications within the past 12 months. In this sample, 58% of responses who took prescription medications within the past 12 months were women and 42% were men. Then, we created this outcome variable by using the four variables for those who took prescription medications within the past 12 months, including “Skipped medication doses to save money, past 12 months”, “Took less medication to save money, past 12 months”, “Delayed filling prescription to save money, past 12 months”, and “Needed prescription medication but did not receive it due to cost, past 12 months” in the 2023 NHIS. People who reported no to all four questions were considered as taking full prescription medications without delay within the past 12 months, coded as 0; people who reported yes to any of the four questions were considered as not taking full prescription medications or with delay within the past 12 months, coded as 1.

#### 2.3.2. Explanatory Variables

Sexual orientation: The 2023 NHIS asked an individual if they think of themselves as gay or lesbian, straight, bisexual, or something else. This variable was recoded with the choices being as follows: straight, coded as 0, and LGB, coded as 1. The categories of gay or lesbian, bisexual, and something else were combined to LGB. Participants who answered “something else” were asked a follow-up question about what they meant by “something else”; however, these responses were not available in the public-use file [30]. We included “something else” in the LGB category to be more inclusive about the LGB definition rather than categorizing people who do not identify their sexual orientation as only straight/heterosexual.

Sex: The 2023 NHIS asked an individual what their identified sex is, with binary choices: male, coded as 0, and female, coded as 1.

Therefore, the present study examines four inter-categorical groups by interacting sexual orientation and sex: LGB women, straight women, LGB men, and straight men, with lesbian and bisexual women as LGB women and gay and bisexual men as LGB men.

Everyday discrimination: For each of the five items mentioned in Section 2.2, participants were given five answer options: at least once a week (coded as 5); a few times a month (coded as 4); a few times a year (coded as 3); less than once a year (coded as 2); and never (coded as 1). Extant studies using similar discrimination items have often applied the mean score of those discrimination items and used the mean score as a continuous explanatory or outcome variable in their models [7,43,44,45]. Following the suit, we calculated the mean score of the five items for each participant and included the mean score as a continuous variable in the model. To gain more nuances of discrimination structural issues, we also included discrimination individual items in a separate model. For each individual item, if the value is larger than or equal to 3, the participant is coded as experiencing a higher level of that everyday discrimination (e.g., being treated with less courtesy or respect); if the value is less than or equal to 2, the participant is coded as experiencing a lower level of that everyday discrimination (e.g., being treated with less courtesy or respect). The rationale behind this coding is that first, the dichotomization of an individual discrimination item is commonly used in perceived discrimination studies [34,46,47]; second, the extant studies often code experiencing any discrimination within the last 12 months or overall as indicating perceived discrimination [34,46,47]; thus, it is reasonable to code “at least once a week”, “a few times a month”, and “a few times a year” as experiencing a higher level of discrimination.

Anticipated discrimination: For each of the four items mentioned in Section 2.2, participants were given five answer options: at least once a week (coded as 5); a few times a month (coded as 4); a few times a year (coded as 3); less than once a year (coded as 2); and never (coded as 1). Following the same rationale as that for everyday discrimination as described in the above paragraph, we calculated the mean score of the four items for each participant and included the mean score as a continuous variable in the model. We also included discrimination individual items in a separate model. For each individual item, if the value is larger than or equal to 3, the participant is coded as experiencing a higher level of that anticipated discrimination (e.g., preparing for possible insults before leaving home); if the value is less than or equal to 2, the participant is coded as experiencing a lower level of that everyday discrimination (e.g., preparing for possible insults before leaving home).

#### 2.3.3. Other Covariates

Age: The age range was filtered to 26 to 64. This variable was recoded with two groups: 26–44, coded as 0, and 45–64, coded as 1. The rationale for the cutoff for the binary age categories is that, first, as mentioned earlier, the expectation of regular check-ups is different for people who are under age 45 and those who are 45 or older [42]; second, existing research on prescription medications also used the same age cutoff (e.g., [33]).

Ethnicity: We restricted the sample to Non-Hispanic White only (NH White, hereafter) (coded as 0), Hispanic (coded as 1), Non-Hispanic Black/African American only (NH Black) (coded as 2), and Non-Hispanic Asian only (NH Asian) (coded as 3). Due to insufficient sample size, we did not include Non-Hispanic American Indian or Alaskan Native (AIAN) only, Non-Hispanic AIAN and any other group, and other single and multiple races in our analysis and recoded them as “NA”.

Marital status: This variable measures an individual’s response to the question about their current marital status. A participant who answered “Married” was coded as 0; a participant who answered, “Living with a partner together as an unmarried people” or “Neither” was combined to “Not Married”, coded as 1.

Education: The 2023 NHIS asked an individual’s education level. This variable was recoded with the five categories: advanced degree, coded as 0; bachelor’s degree, coded as 1; some college or associate degree, coded as 2; high school or equivalent, coded as 3; and less than high school, coded as 4.

Poverty status: The 2023 NHIS provided this variable that describes the ratio of family income to poverty threshold for an adult’s family. The 2023 NHIS used the U.S. Census Bureau’s weighted average poverty thresholds for 2021 (the 2022 poverty thresholds were not available when the 2023 NHIS instrument was created) relative to family size and calculated the ratio using the self-reported and imputed family income (23.7% missing family income) divided by the corresponding poverty threshold [35]. The 2023 NHIS used multiple imputation technology to impute missing data on the family income variable. This complex technology is reported in the document “Multiple Imputation of Family Income in 2023 NHIS: Methods” [48]. If the ratio is equal to or greater than 1, we categorized it as “Not in Poverty”, coded as 0; if the ratio is less than 1, we categorized it as “In Poverty”, coded as 1.

Urban–rural classification: This variable measures an individual’s county as rural or urban. “Large central metro”, “Large fringe metro”, and “Medium and small metro” were combined to “Urban”, coded as 0, and “Nonmetropolitan” was categorized as “Rural”, coded as 1.

Insurance coverage source: We created this variable to cover private, public, and other insurance. The 2023 NHIS asked about health insurance type, and the answer options included “Private”, “Medicaid and other public”, and “Other coverage”. For people who had private insurances, the 2023 NHIS also asked how they obtained their health insurance plan. After we combined these two variables, a participant who answered “Through an employer, union, or professional organization” was coded as 0; a participant who answered “Purchased directly”, “Through Healthcare.gov or the Affordable Care Act”, “Through a state or local government or community program”, “Other”, “Through school”, “Through parents”, or “Through other relative” was combined to “Others”, coded as 1; and a participant who answered “Medicaid and other public” or “Other coverage”, coded as 2.

Deductible: We created this variable to cover the deductible information for private and Medicaid insurance. For people who had private or Medicaid insurance, the 2023 NHIS asked if an individual’s health insurance plan has an annual deductible. After we combined these two variables, and since there is a substantial number of people who reported “Don’t know”, a participant who answered “No” was coded as 0, “Don’t know” coded as 1, and “Yes” coded as 2. The 2023 NHIS did not provide the deductible information of another major insurance–Medicare, but Medicare is mostly for people who are 65 or older, out of the age range of this research, and its deductible is complicated and varies depending on different plans and different types of visits (https://www.ncoa.org/article/what-is-the-medicare-deductible/, accessed on 3 December 2025), so missing this information would not significantly impact our results.

Difficulty paying medical bills: This variable measures an individual’s response to the question about medical-related payment issues within the past 12 months. A participant who answered “No” was coded as 0; a participant who answered “Yes” was coded as 1.

Having a usual place for care: The 2023 NHIS asked if an individual has a place that they usually go to if they are sick and need healthcare. A participant who answered “Yes” or “There is more than one place” was combined with “Yes”, coded as 0; a participant who answered “There is no place” was coded as 1.

Transportation barrier: The 2023 NHIS asked if an individual has had transportation barriers to medical appointments, meetings, work, and other necessary daily living things within the past 12 months. A participant who answered “No” was coded as 0 and “Yes” coded as 1.

Self-rated health: This variable measures an individual’s self-rated general health. “Excellent”, “Very good”, or “Good” was combined with “Better Status”, coded as 0; “Poor” or “Fair” was coded as 1.

### 2.4. Statistical Methodology

The present study followed the steps recommended for quantitative intersectional analysis for logistic regression using complex survey data [27,31,33].

First, we fit a series of logistic regression models with sexual orientation–sex interactions for each routine healthcare utilization/access outcome, respectively. Model 1 included Everyday Discrimination mean score as the structural variable; Model 2 included Everyday Discrimination individual items; Model 3 included Anticipated Discrimination mean score; and Model 4 included Anticipated Discrimination individual items. All the models adjusted for age, ethnicity, marital status, education, poverty status, urban–rural classification, insurance coverage source, deductible, difficulty paying medical bills, having a usual place for care, transportation barrier, and self-rated health. We included Everyday Discrimination and Anticipated Discrimination as the structural variables separately in the models. Anticipated Discrimination may mediate the association between Everyday Discrimination and healthcare utilization/access; however, this mediation analysis would be the subject of another paper. As the first step to examine the association between social structure and routine healthcare utilization/access, creating models including Everyday Discrimination and Anticipated Discrimination, respectively, helps provide a novel and full picture of such a relationship. Additionally, when including all the Everyday Discrimination individual items in the same model and all the Anticipated Discrimination individual items in the same model, we tested for multicollinearity using the variance inflation factor (VIF), and no multicollinearity issues were detected.

Second, we calculated average marginal predictions for inter-categorical groups by interacting sexual orientation and sex and other covariates, accounting for the complex survey design (weighting, clustering, and stratification).

Third, we computed risk ratios of average marginal predictions for all the covariates.

**Figure 1:**
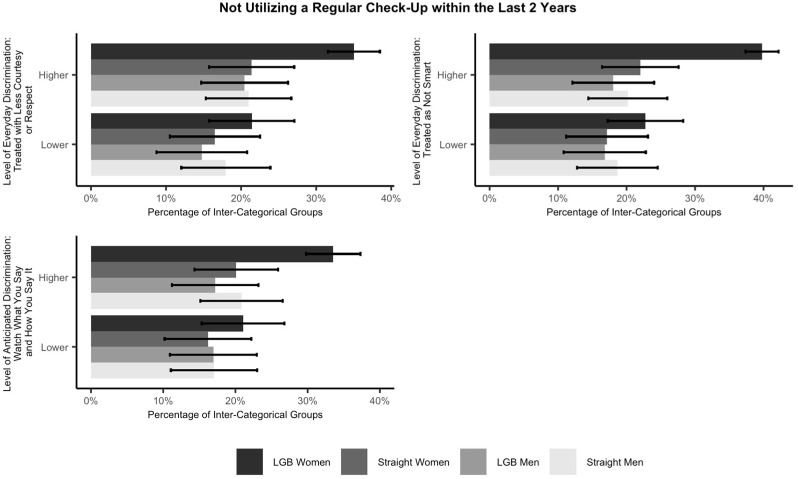
Percentage of not utilizing a regular check-up within the last 2 years across inter-categorical groups and levels of discrimination with 95% confidence intervals. Note. This figure only includes statistically significant discrimination variables from the models.

**Figure 2:**
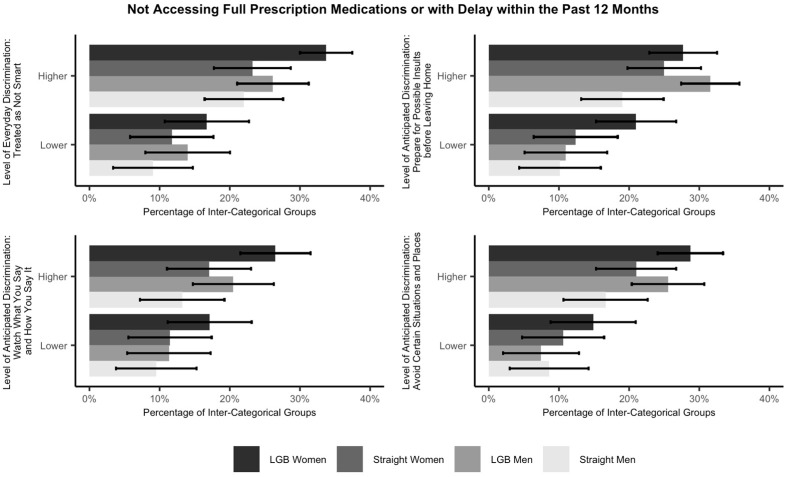
Percentage of not accessing full prescription medications or with delay within the past 12 months across inter-categorical groups and levels of discrimination with 95% confidence intervals. Note. This figure only includes statistically significant discrimination variables from the models.

Lastly, we included the interaction of inter-categorical groups and structural variables (to test Hypothesis 3 but only including the statistically significant discrimination variables from the previous step). Due to the small sample sizes of some groups (e.g., LGB women and LGB men who reported different levels of discrimination; see Table 3) resulting from the three-way interaction (sexual orientation, sex, and discrimination), the power of regression tests including the effect of the three-way interaction on routine healthcare utilization/access will be significantly reduced. Therefore, we alternatively conducted descriptive analyses to examine this matter. First, we used the original data to create a descriptive plot to illustrate the percentage of not utilizing/accessing healthcare across inter-categorical groups and different levels of discrimination (see the results in Figure 1 and Figure 2).

Further, we examined if experiencing a higher level of discrimination would have a stronger effect on LGB people than their straight peers with respect to not utilizing/accessing routine healthcare. To do so, we compared mean differences in routine healthcare utilization/access of LGB and straight people experiencing different levels of discrimination. We used the following technique demonstrated in extant research [49,50] to handle unequal group sizes because unequal group sizes reduce the power of statistical tests in terms of the ability to detect mean differences across groups. For each statistically significant discrimination variable from the models, we created four equally sized groups of responses, respectively: LGB people who experience a higher level of discrimination; straight people who experience a higher level of discrimination; LGB people who experience a lower level of discrimination; and straight people who experience a lower level of discrimination. Then, we selected a random sample of the size of the smallest group from each of the remaining three groups, creating four equally sized groups.

All analyses were completed in R 4.5.0. We tested for multicollinearity for all the models using the VIF; no multicollinearity issues were detected.

## 3. Results

### 3.1. Descriptive Statistics

Table 2 presents the descriptive statistics of our weighted sample. For regular checkups, most (94.4%) of the sample are straight people; 5.6% are LGB people. Males account for 48.7% of the sample and females account for 51.3%. Regarding inter-categorical groups, 48.1% of the sample are straight women, 46.5% are straight men, 3.2% are LGB women, and 2.3% are LGB men. Regarding structural variables, the mean value of the average scores of everyday discrimination is 1.6 (ranging from 1 to 5, with higher values being higher levels of discrimination), whereas the mean value of the average scores of anticipated discrimination is 1.98. A total of 81.5% of the sample had a wellness visit, physical, or general-purpose check-up within the past 2 years, and 18.5% did not.

For prescription medications, the percentage distributions are similar. A total of 93.9% of the sample are straight people; 6.1% are LGB people. Males account for 45.1% of the sample and females account for 54.9%. Regarding inter-categorical groups, 51.3% of the sample are straight women, 42.7% are straight men, 3.6% are LGB women, and 2.4% are LGB men. Regarding structural variables, the mean value of the average scores of everyday discrimination is 1.62, whereas the mean value of the average scores of anticipated discrimination is 2.01. A total of 87.5% of the sample had full prescription medications without delay within the past 12 months, and 12.5% did not.

### 3.2. Findings from Regression Analysis

Table 4 presents the average marginal predictions in percentage for the four inter-categorical groups by interacting sexual orientation and sex. They are the estimated percentages of not having a wellness visit, physical, or general-purpose check-up within the last 2 years, adjusting for model covariates. The second column includes Everyday Discrimination mean score as the structural variable in the model; the third column includes Everyday Discrimination individual items; the fourth column includes Anticipated Discrimination mean score; and the fifth column includes Anticipated Discrimination individual items. Regarding Everyday Discrimination, the estimated percentage of not utilizing a wellness visit, physical, or general-purpose check-up is the highest for LGB women, then LGB men, straight men, and straight women. There is a statistically significant difference between LGB women and straight women. When adjusting for Everyday Discrimination mean score, there is also a statistically significant difference between LGB women and straight men. Regarding Anticipated Discrimination, the estimated percentage of not utilizing a wellness visit, physical, or general-purpose check-up is the highest for LGB women, then straight men, LGB men, and straight women. There is a statistically significant difference between LGB women and straight women as well as between LGB women and straight men.

For not having full prescription medications without delay within the past 12 months, Table 5 presents the average marginal predictions in percentage for the four inter-categorical groups by interacting sexual orientation and sex, adjusting for model covariates. Similarly to Table 4, the second column includes Everyday Discrimination mean score as the structural variable in the model; the third column includes Everyday Discrimination individual items; the fourth column includes Anticipated Discrimination mean score; and the fifth column includes Anticipated Discrimination individual items. The results in those columns are close. The estimated percentage of not having full prescription medications without delay is the highest for LGB men, then LGB women, straight women, and straight men. There is a statistically significant difference between LGB men and straight men.

Table 6 presents the risk ratios of average marginal predictions from the logistic regression models of healthcare utilization of a wellness visit, physical, or general-purpose check-up within the last 2 years. Model 1 includes Everyday Discrimination mean score as the structural variable; Model 2 includes Everyday Discrimination individual items; Model 3 includes Anticipated Discrimination mean score; and Model 4 includes Anticipated Discrimination individual items. All the models adjust for age, ethnicity, marital status, education, poverty status, urban–rural classification, insurance coverage source, deductible, difficulty paying medical bills, having a usual place for care, transportation barrier, and self-rated health. In the supplemental materials, Table S1 shows Models 1–4 with all the covariates. Here, only the intersectional groups and the structural variable in each model, respectively, are shown. For all the models except Model 2, LGB women are statistically significantly (over 1.2 times) more likely to not utilize a wellness visit, physical, or general-purpose check-up compared to straight men (the reference group). For all the models, there are no statistically significant differences between straight women and straight men or LGB men and straight men. Moreover, people who reported a higher Everyday Discrimination mean score are statistically significantly (1.15 times) more likely to not utilize a wellness visit, physical, or general-purpose check-up with the last 2 years compared to those who reported a lower Everyday Discrimination mean score. Regarding Everyday Discrimination individual items, people who reported experiencing a higher level of being treated with less courtesy or respect (1.17 times) or being treated as not smart (1.16 times) are statistically significantly more likely to not utilize a wellness visit, physical, or general-purpose check-up with the last 2 years compared to those who reported experiencing a lower level of being treated with less courtesy or respect or being treated as not smart, respectively. Similarly, people who reported a higher Anticipated Discrimination mean score are statistically significantly (1.12 times) more likely to not utilize a wellness visit, physical, or general-purpose check-up with the last 2 years compared to those who reported a lower Anticipated Discrimination mean score. Regarding Anticipated Discrimination individual items, people who reported experiencing a higher level of “watching what you say and how you say it” (1.15 times) are statistically significantly more likely to not utilize a wellness visit, physical, or general-purpose check-up with the last 2 years compared to those who reported experiencing a lower level of “watching what you say and how you say it”.

Table 7 presents the risk ratios of average marginal predictions from the logistic regression models of healthcare access to full prescription medications without delay within the past 12 months. Model 1 includes Everyday Discrimination mean score as the structural variable; Model 2 includes Everyday Discrimination individual items; Model 3 includes Anticipated Discrimination mean score; and Model 4 includes Anticipated Discrimination individual items. All the models adjust for age, ethnicity, marital status, education, poverty status, urban–rural classification, insurance coverage source, deductible, difficulty paying medical bills, having a usual place for care, transportation barrier, and self-rated health. In the Supplementary Material, Table S2 shows Models 1–4 with all the covariates. Here, only the intersectional groups and the structural variable in each model, respectively, are shown. For all the models, LGB women are statistically significantly (over 1.5 times) more likely to not have full prescription medications or with delay within the past 12 months compared to straight men (the reference group); LGB men (over 1.6 times) and straight women (over 1.3 times) are also statistically significantly more likely to report the event compared to straight men. Moreover, people who reported a higher Everyday Discrimination mean score are statistically significantly (1.36 times) more likely to not have full prescription medications or with delay within the past 12 months compared to those who reported a lower Everyday Discrimination mean score. Regarding Everyday Discrimination individual items, people who reported experiencing a higher level of being treated as not smart (1.34 times) are statistically significantly more likely to not have full prescription medications or with delay within the past 12 months compared to those who reported experiencing a lower level of being treated as not smart. Similarly, people who reported a higher Anticipated Discrimination mean score are statistically significantly (1.3 times) more likely to not have full prescription medications or with delay within the past 12 months compared to those who reported a lower Anticipated Discrimination mean score. Regarding Anticipated Discrimination individual items, people who reported experiencing a higher level of preparing for possible insults before leaving home (1.23 times), “watching what you say and how you say it” (1.24 times), or avoiding certain situations and places (1.29 times) are statistically significantly more likely to not have full prescription medications or with delay within the past 12 months compared to those who reported experiencing a lower level of those anticipated discriminations, respectively.

To examine the effect of structural variables on LGB people, the interaction of inter-categorical groups and discrimination is considered. As mentioned in the Statistical Methodology section, due to the small sample sizes of some groups (e.g., LGB women and LGB men who reported different levels of discrimination) resulting from the three-way interaction (sexual orientation, sex, and levels of discrimination), the power of logistic regression tests including the effect of the three-way interaction on routine healthcare utilization/access will be significantly reduced. Therefore, we conducted descriptive analyses to examine this matter. Using the original data, Figure 1 and Figure 2 illustrate the percentage of not utilizing/accessing routine healthcare across inter-categorical groups and levels of discrimination. Among the inter-categorical groups, LGB women who experience a higher level of discrimination (i.e., being treated with less courtesy or respect, being treated as not smart, or “watching what you say and how you say it”) have the highest percentage (over 30%) of not utilizing a wellness visit, physical, or general-purpose check-up within the last 2 years, whereas both LGB men and LGB women who experience a higher level of discrimination (i.e., being treated as not smart, preparing for possible insults before leaving home, “watching what you say and how you say it”, or avoiding certain situations and places) have the highest percentages (over 20%) of not accessing full prescription medications or with delay within the past 12 months.

Further, Table 8 (see columns indicating LGB–Straight w/Higher Level of Discrimination) compares the mean differences in utilizing regular check-ups between LGB people who experience a higher level of discrimination (i.e., being treated with less courtesy or respect, being treated as not smart, or “watching what you say and how you say it”) and straight people who experience a higher level of discrimination. LGB people who experience a higher level of discrimination are statistically significantly less likely to utilize regular check-ups as compared to straight people who experience a higher level of discrimination. This result implies that experiencing a higher level of discrimination has a stronger effect on LGB people than straight people with respect to not utilizing a regular check-up within the last 2 years.

Similarly, Table 9 (see columns indicating LGB–Straight w/Higher Level of Discrimination) compares the mean differences in accessing prescription medications between LGB people who experience a higher level of discrimination (i.e., being treated as not smart, preparing for possible insults before leaving home, “watching what you say and how you say it”, or avoiding certain situations and places) and straight people who experience a higher level of discrimination. LGB people who experience a higher level of discrimination are statistically significantly less likely to access prescription medications as compared to straight people who experience a higher level of discrimination. This result implies that experiencing a higher level of discrimination has a stronger effect on LGB people than straight people with respect to not accessing full prescription medications or with delay within the past 12 months.

## 4. Discussion

### 4.1. LGB Women Are Least Likely to Utilize Regular Check-Ups and LGB Men Are Least Likely to Access Prescription Medications Among the Inter-Categorical Groups by Interacting Sexual Orientation and Sex

In partial support of Hypothesis 1, our quantitative intersectional analysis shows that among the inter-categorical groups by interacting sexual orientation and sex, LGB women are least likely to have a wellness visit, physical, or general-purpose check-up within the last 2 years (Table 6), whereas LGB men are least likely to have full prescription medications without delay within the past 12 months (Table 7), after controlling for all the covariates. Regarding regular check-ups, for all the models except Model 2 (Table 6), LGB women are statistically significantly less likely to utilize regular check-ups compared to straight men. There are no statistically significant differences between straight women and straight men or LGB men and straight men (Table 6). Regarding prescription medications, for all the models (Table 7), LGB men are statistically significantly less likely to access prescription medications compared to straight men. LGB women and straight women are also less likely to access prescription medications as compared to straight men (Table 7). Our findings are consistent with existing research that LGB women are less likely to utilize regular check-ups compared to straight women, straight men, or LGB men. Unlike LGB men who have targeted HIV/AIDS care services, LGB women do not have targeted healthcare programs [2]. Compared to straight people, driven by minority and social structure factors, LGB women are less likely to seek regular check-ups [2]. Additionally, masculine gender role norms may discourage straight men from seeking healthcare, reducing the utilization gaps between LGB men and straight men, while no similar ideology applies to the utilization inequities between LGB women and straight women [2,10].

Also echoing existing research, LGB men are least likely to access prescription medications among the inter-categorical groups by interacting sexual orientation and sex, while LGB women and straight women are also less likely to access prescription medications than straight men. Research on prescription medication use for people who have chronic diseases suggests similar results that straight men with chronic diseases are most likely to have filled all prescribed medications in comparison to LGB women and men with chronic diseases [51]. One possible explanation is LGB people’s socioeconomic disadvantages and higher rates of being uninsured for prescription medications [51,52]. The reasons why LGB men are least likely to access prescription medications among the inter-categorical groups by interacting sexual orientation and sex are unclear. Our results suggest that policies can target LGB women, offering more lesbian/bisexual women-affirmative care resources and raising awareness of potential intersectional sex and sexual minority oppressions LGB women have experienced. Regarding prescription medication, policies can target LGB men, LGB women, and straight women, providing training to healthcare professionals to increase their awareness of sexual orientation, gender identity, and discrimination effects on healthcare access inequities.

From a methodological perspective, the present study applies quantitative intersectional analysis for logistic regression based on complex survey data. Drawing from Bieler et al.’s [33] approach using average marginal predictions and following the recommendation of using risk ratios for quantitative intersectional analysis for logistic regression [27], we first calculate average marginal predictions for inter-categorical groups defined by sexual orientation and sex, adjusting for model covariates. The average marginal predictions are used to compare the estimated percentages of not utilizing/accessing routine healthcare among the four inter-categorical groups. This is important for quantitative intersectional analysis of complex survey data because, first, it is recommended to, where possible, make intersectional inequities visible by starting with a descriptive intersectional analysis to ensure levels of outcomes for those at particular intersections are not obscured [27,53]; second, one of the goals of analyzing complex survey data is to produce descriptive statistics for the population that is as important as subsequent estimation of risk ratios [33]. Then, we compute risk ratios of average marginal predictions from the logistic regression models. For quantitative intersectional analysis for log-scale regression such as logistic regression, it is recommended to compute risk ratios instead of odds ratios for easier and clearer interpretations of intersectional results and more targeted and actionable policy suggestions [27,31]. Our study is among the first to systematically and comprehensively apply these quantitative intersectional methods in empirical studies to show their helpfulness.

### 4.2. Experiencing a Higher Level of Discrimination Is Positively Associated with Lower Utilization of and Access to Routine Healthcare and This Effect Is Stronger for LGB People

In support of Hypothesis 2, the current analysis shows that people who report experiencing a higher level of discrimination are less likely to utilize/access routine healthcare than those who report experiencing a lower level of discrimination. For regular check-ups, this is true for Everyday Discrimination and Anticipated Discrimination mean scores, two Everyday Discrimination individual items including being treated with less courtesy or respect and being treated as not smart, and one Anticipated Discrimination individual item “watching what you say and how you say it”, when adjusting for all the model covariates (Table 6). For prescription medications, this is true for Everyday Discrimination and Anticipated Discrimination mean scores, one Everyday Discrimination individual item including being treated as not smart, and three Anticipated Discrimination individual items including preparing for possible insults before leaving home, “watching what you say and how you say it”, and avoiding certain situations and places, when adjusting for all the model covariates (Table 7).

For the statistically significant discrimination individual items, we further investigate the interaction of inter-categorical groups/sexual orientation and discrimination. Due to the small sample sizes of some groups (e.g., LGB women and LGB men who reported different levels of discrimination) resulting from the three-way interaction (sexual orientation, sex, and discrimination), we were unable to conduct logistic regression models including the three-way interaction because the power of the regression tests would be significantly reduced. This is an important finding regarding data collection. Understanding the intersectional and structural issues of healthcare utilization/access inequities for LGB people is a holistic process and requires more adequate data collection of LGB men and LGB women.

Alternatively, we conducted descriptive analyses to examine the three-way interaction. In support of Hypothesis 3, as shown in Table 8 and Table 9, we find that regarding regular check-ups, reporting experiencing a higher level of discrimination (i.e., being treated with less courtesy or respect, being treated as not smart, or “watching what you say and how you say it”) has a stronger effect on LGB people than their straight peers. Regarding prescription medications, reporting experiencing a higher level of discrimination (i.e., being treated as not smart, preparing for possible insults before leaving home, “watching what you say and how you say it”, or avoiding certain situations and places) also has a stronger effect on LGB people than straight people.

This study is a critical first step to understand social structures attributed to healthcare utilization/access inequities of LGB people. It is important to understand discrimination from a structural level and how that might influence LGB people’s healthcare utilization/access. Related to discrimination, the theory of stigma, as people who are stigmatized often experience discrimination, suggests that people who are stigmatized develop the feeling of stigmatization based on social interactions [21,22,23]. This feeling of being devalued might persist within the healthcare system when those who are stigmatized seek medical services [22,23,54]. Some studies indicate that LGB people often report that fear of being stigmatized is one of the barriers to seeking health services [22,36]. Reviewing the statistically significant discrimination individual items we found (i.e., being treated with less courtesy or respect, being treated as not smart, preparing for possible insults before leaving home, “watching what you say and how you say it”, and avoiding certain situations and places), they are all relevant to the fear of being stigmatized or devalued. For people who experienced everyday discrimination such as being treated with less courtesy or respect or being treated as not smart, they may transform such negative experiences to fear of being stigmatized or devalued in the healthcare setting. For people who experienced anticipated discrimination, their fear of being stigmatized or devalued may persist within the healthcare system, especially; the question of “avoid certain situations and places” identifies hospitals as one of the places in its prompt.

The stronger effect of experiencing a higher level of discrimination on lower utilization of and access to routine healthcare for LGB people than their straight counterparts (straight men regarding prescription medications) is consistent with Schmitt et al.’s [54] argument, stating “when privileged group members are rejected by the disadvantaged... it carries fewer implications for the in-group’s value and status within the culture as a whole” (p. 199). This means that discrimination takes less of a toll on straight people because they have the socially privileged sexual identity, but on the other hand, the effect of discrimination is more detrimental for LGB people since discrimination is connected with culturally and institutionally supported inequities LGB people have experienced because of their sexual orientation [34]. LGB people are more vulnerable to discrimination and less likely to utilize/access routine health services. Furthermore, regarding regular check-ups, there is a lack of supportive health services for LGB people that may cause mistrust between LGB people and providers, resulting in LGB people’s underutilization of routine health services [4,5,55]. First, it is challenging to obtain appointments with specialists for LGB people [2,56]. Second, several studies reported that providers lack training on LGB health issues, are culturally incompetent and not sensitive around LGB health issues, and are uncomfortable with and afraid of treating LGB patients [1,4,41,57]. Regarding prescription medications, our findings confirm that cost reasons partially explain healthcare access inequities [3,4,5]. Our results also imply that LGB people may experience inequities in different aspects of their lives such as employment, income, healthcare resources, and housing [55], so when they experience prescription medication affordability issues, they are more likely to choose to spend their resources on other necessities instead of figuring out prescription medications. This may also suggest that the discrimination structural variable we are testing in our study may play a role in this as discrimination systematically affects LGB people’s daily lives and may be associated with their lack of a variety of resources including healthcare resources. These structural issues are problematic for LGB people who have chronic diseases because routine check-ups and taking prescription medications at the right time are essential to them. These inequities need to be addressed in order to improve LGB people’s healthcare utilization/access. Healthcare policies should focus on increasing supportive resources and services for LGB people.

### 4.3. Limitations

Although our study delves deeper into intersectional and structural issues of routine healthcare utilization and access inequities for LGB people, it does have its limitations. First, the NHIS’s approach to gender was a limitation, as it only recognized sex assigned at birth: male and female. This binary perspective restricts the applicability of our findings to the full spectrum of gender identities and also inherently overlooks the diversity of gender experiences. Additionally, there was no option for respondents who identified as male or female to signify whether they were cisgender or transgender. The restriction to a binary sex classification hinders the comprehensive understanding of how diverse identities may influence an individual’s healthcare utilization and access. This part of our research cannot accurately capture the experiences of non-binary and other trans-identifying individuals; thus, the generalizability of our findings is limited. Because of this, future research would benefit from a more inclusive approach to gender, encompassing a broader range of identities beyond the male/female binary. Recognizing this broad range of gender identities in data collection would offer a more accurate representation of the population. Thus, the survey questions of the NHIS would benefit from the inclusion of the diverse experiences of various individuals. Furthermore, our analysis could be benefited if there were more sufficient sample sizes of some inter-categorical groups (e.g., LGB men and LGB women). Further data collection should adopt an intersectional lens by oversampling sexual and gender minorities. Future research can also conduct path analysis to examine how discrimination affects healthcare utilization and access through other variables (e.g., difficulty paying medical bills, health status). Moreover, although it is important to understand the association between discrimination outside the healthcare system and healthcare utilization/access, discrimination within the healthcare system was not analyzed because the NHIS lacks the required measures. Collecting data on discrimination both outside and within the healthcare system in the same survey can help conduct more analyses such as causal inference and build a more comprehensive understanding of the association between social structure and healthcare utilization/access. Furthermore, the discrimination scales are unable to distinguish whether discrimination is due to one’s LGB identity or other reasons, so even though we conducted the analyses on the interaction of sexual orientation and discrimination, there may be other unknown factors that can explain discrimination. Also, the current study is the first step to understanding the intersectional and structural issues of routine healthcare utilization and access for LGB people. Future studies can explore more outcome variables of healthcare utilization/access, such as urgent care/emergency rooms use and mental healthcare utilization. Lastly, we used the self-reported data on sexual orientation and structural variables (Everyday Discrimination and Anticipated Discrimination), and it may have response biases. Participants may be more concerned with their tendency to align with perceived expectations rather than their accurate lived experiences; thus, the accuracy of the data is at stake. Future research could benefit from exploring other surveys and link different survey data sources, if possible, to explore a more holistic analysis.

## 5. Conclusions

The present study contributes to the health services literature by empirically examining the association between social structure and routine healthcare utilization/access for LGB people with chronic diseases and applying quantitative intersectional methods for logistic regression to understand routine healthcare utilization/access from a sexual orientation and sex intersectional lens. We innovatively include Everyday Discrimination and Anticipated Discrimination as structural variables to explore the association between perceived discrimination outside the healthcare system and routine healthcare utilization/access. Our results indicate that experiencing a higher level of discrimination is positively associated with lower utilization of regular check-ups and lower access to prescription medications. Regarding regular check-ups, reporting experiencing a higher level of discrimination (i.e., being treated with less courtesy or respect, being treated as not smart, or “watching what you say and how you say it”) has a stronger effect on LGB people than their straight peers. Regarding prescription medications, reporting experiencing a higher level of discrimination (i.e., being treated as not smart, preparing for possible insults before leaving home, “watching what you say and how you say it”, or avoiding certain situations and places) also has a stronger effect on LGB people than straight people. Highlighting structural issues of healthcare utilization/access offers new evidence on healthcare utilization/access inequities that can inform policies for raising awareness of and addressing structural issues. Additionally, we demonstrate how to implement several steps recommended for quantitative intersectional analysis for logistic regression using complex survey data: computing average marginal predictions and risk ratios for easier and clearer interpretations of intersectional results and more actionable policy implications for inter-categorical groups. Our results show that among the inter-categorical groups by interacting sexual orientation and sex, LGB women are least likely to utilize regular check-ups and LGB men are least likely to access prescription medications and suggest that relevant policies target LGB women and LGB men.

## Figures and Tables

**Table 1:** A made-up example to illustrate the advantages of risk ratios over odds ratios.

	Not Using Regular Check-Ups Within the Last 2 Years	Using Regular Check-Ups Within the Last 2 Years
LGB Individuals	77	23
Straight Individuals	64	36
Risk Ratio: $\frac{77/100}{64/100}$ *≈* 1.2, Interpretation: LGB people are about 1.2 times as likely to not use regular check-ups within the last 2 years as straight people.		
Odds Ratio: $\frac{77/23}{64/36}$ *≈* 1.9, Interpretation: The odds of LGB people not using regular check-ups within the last 2 years are about 1.9 times higher than those of straight people.		

**Table 2:** Descriptive statistics of the sample (Weighted. Total *N* = 136,231,053 for regular check-ups; total *N* = 97,786,893 for prescription medications).

	Regular Check-Ups		Prescription Medications	
Variable	n	%	n	%
Sexual Orientation				
Straight	125,369,049	94.4	89,530,671	93.9
LGB	7,468,699	5.6	5,852,957	6.1
Sex				
Male	66,345,122	48.7	44,090,969	45.1
Female	69,857,156	51.3	53,667,148	54.9
Inter-Categorical Groups				
Straight Men	63,267,171	46.5	41,768,107	42.7
LGB Men	3,077,951	2.3	23.2	2.4
Straight Women	65,481,413	48.1	50,152,058	51.3
LGB Women	4,375,743	3.2	3,515,090	3.6
Age				
25–44	66,292,803	48.7	42,697,088	43.7
45–64	69,938,250	51.3	55,089,805	56.3
Ethnicity				
NH White	84,441,716	63.7	63,720,668	66.8
Hispanic	23,200,111	17.5	14,720,693	15.4
NH Black	16,604,838	12.5	11,714,936	12.3
NH Asian	8,369,706	6.3	5,163,364	5.4
Marital Status				
Married	78,680,690	58.6	57,725,398	59.9
Not married	55,533,468	41.4	38,650.795	40.1
Everyday Discrimination (Mean Score)				
Mean value ^a^	1.60		1.62	
Anticipated Discrimination (Mean Score)				
Mean value ^a^	1.98		2.01	
Education				
Advanced Degree	19,917,066	14.7	14,867,264	15.3
Bachelor’s Degree	31,589,441	23.3	22,805,578	23.4
Some College/Associate Degree	39,108,066	28.8	28,141,338	28.9
High School/Equivalent	33,237,697	24.5	23,486,319	24.1
Less than High School	11,744,064	8.7	8,083,397	8.3
Poverty Status				
Not in Poverty	123,690,586	90.8	88,948,248	91.0
In Poverty	12,540,468	9.2	8,838,645	9.0
Urban–Rural Classification				
Urban	117,895,941	86.5	84,079,588	86.0
Rural	18,336,112	13.5	13,707,305	14.0
Insurance Coverage Source				
Private Insurances: Employer/Union/Professional Organization	82,494,289	68.9	60,603,158	67.8
Private Insurances: Others	10,308,905	8.6	7,503,643	8.4
Public/Other Insurances	27,013,838	22.5	21,253,696	23.8
Deductible				
No	34,327,724	29.6	25,769,621	30.0
Do Not Know	6,139,136	5.3	3,972,730	4.6
Yes	75,491,579	65.1	56,087,274	65.3
Difficulty Paying Medical Bills				
No	117,953,953	86.6	83,136,340	85.0
Yes	18,277,100	13.4	14,659,553	15.0
Having a Usual Place for Care				
No	122,082,079	89.7	92,292,368	94.4
Yes	14,091,889	10.3	5,459,105	5.6
Transportation Barrier				
No	123,465,609	92.7	88,259,692	92.3
Yes	9,719,892	7.3	7,328,150	7.7
Self-rated Health				
Better Status	115,383,726	84.7	79,899,127	81.7
Poor/Fair	20,798,112	15.3	17,844,821	18.3
Routine Healthcare Utilization: Wellness Visit, Physical, or General-Purpose Check-up within the Last 2 Years				
Yes	101,655,697	81.5	-	-
No	23,143,979	18.5	-	-
Routine Healthcare Access: Full Prescription Medications without Delay within the Past 12 Months				
Yes	-	-	85,598,336	87.5
No	-	-	12,188,556	12.5

Note. ^a^ The mean value of the average scores of everyday discrimination and anticipated discrimination ranges from 1 to 5, with higher values being higher levels of discrimination.

**Table 3:** Distributions of the three-way interaction groups (unweighted).

Discrimination	Straight				LGB			
	Men	Row Total %	Women	Row Total %	Men	Row Total %	Women	Row Total %
Higher Level of Everyday Discrimination: Treated with Less Courtesy or Respect	2037	40.4	2571	51.1	136	2.7	292	5.8
Lower Level of Everyday Discrimination: Treated with Less Courtesy or Respect	4336	44.9	4896	50.7	203	2.1	220	2.3
Higher Level of Everyday Discrimination: Treated as Not Smart	1089	36.5	1620	54.3	78	2.6	195	6.5
Lower Level of Everyday Discrimination: Treated as Not Smart	5284	45.1	5847	49.9	261	2.2	317	2.7
Higher Level of Anticipated Discrimination: Watch What You Say and How You Say It	3189	42.3	3802	50.4	211	2.8	335	4.4
Lower Level of Anticipated Discrimination: Watch What You Say and How You Say It	3184	44.5	3665	51.2	128	1.8	177	2.5

Note. This table uses the sample for regular check-ups as an example to illustrate the issue of the small sample sizes of LGB men and LGB women groups. Since the sample for prescription medications is smaller than and has similar distributions to that for regular check-ups, the issue of the small sample sizes remains. In addition, this table only includes statistically significant discrimination variables from the models.

**Table 4:** Estimated percentage of not having a wellness visit, physical, or general-purpose check-up within the last 2 years for inter-categorical groups.

Inter-Categorical Group	Average Marginal Prediction ^1^, % (SE)	Average Marginal Prediction ^2^, % (SE)	Average Marginal Prediction ^3^, % (SE)	Average Marginal Prediction ^4^, % (SE)
LGB Women	23.27 (0.02)	23.27 (0.02)	23.53 (0.02)	23.62 (0.02)
Straight Women	17.67 * (0.01)	17.67 * (0.01)	17.69 * (0.01)	17.67 * (0.01)
LGB Men	18.63 (0.03)	18.63 (0.03)	18.31 (0.03)	18.35 (0.03)
Straight Men	18.52 * (0.01)	18.52 (0.01)	18.49 * (0.01)	18.50 * (0.01)

Note. SE stands for standard error. ^1^ Percentage estimates were adjusted for the Everyday Discrimination mean score and other covariates. ^2^ Percentage estimates were adjusted for the Everyday Discrimination individual items and other covariates. ^3^ Percentage estimates were adjusted for the Anticipated Discrimination mean score and other covariates. ^4^ Percentage estimates were adjusted for the Anticipated Discrimination individual items and other covariates. * indicates a statistically significant difference between LGB women and that group.

**Table 5:** Estimated percentage of not having full prescription medications without delay within the past 12 months for inter-categorical groups.

Inter-Categorical Group	Average Marginal Prediction ^1^, % (SE)	Average Marginal Prediction ^2^, % (SE)	Average Marginal Prediction ^3^, % (SE)	Average Marginal Prediction ^4^, % (SE)
LGB Women	130.54 (0.02)	130.31 (0.02)	130.21 (0.02)	130.00 (0.02)
Straight Women	110.80 (0.01)	110.74 (0.01)	110.76 (0.01)	110.74 (0.01)
LGB Men	150.11 (0.02)	150.07 (0.02)	140.29 (0.02)	140.33 (0.02)
Straight Men	80.43 * (0.01)	80.52 * (0.01)	80.57 * (0.01)	80.58 * (0.01)

Note. SE stands for standard error. ^1^ Percentage estimates were adjusted for the Everyday Discrimination mean score and other covariates. ^2^ Percentage estimates were adjusted for the Everyday Discrimination individual items and other covariates. ^3^ Percentage estimates were adjusted for the Anticipated Discrimination mean score and other covariates. ^4^ Percentage estimates were adjusted for the Anticipated Discrimination individual items and other covariates. * indicates a statistically significant difference between LGB men and that group.

**Table 6:** Logistic regression models of routine healthcare utilization within the last 2 years whether or not one had a wellness visit, physical, or general-purpose check-up (risk ratios (95%CI)).

Variable	Model 1	Model 2	Model 3	Model 4
LGB Women vs. Straight Men	**1.29 (1.02, 1.56)**	1.26 (0.99, 1.52)	**1.27 (1.01, 1.54)**	**1.28 (1.01, 1.55)**
Straight Women vs. Straight Men	0.96 (0.87, 1.05)	0.95 (0.86, 1.04)	0.96 (0.87, 1.05)	0.96 (0.87, 1.04)
LGB Men vs. Straight Men	1.01 (0.71, 1.32)	1.01 (0.71, 1.31)	0.99 (0.69, 1.29)	0.99 (0.69, 1.29)
Everyday Discrimination (Mean Score)	**1.15 (1.08, 1.22)**			
Everyday Discrimination: Treated with Less Courtesy or Respect (Higher vs. Lower)		**1.17 (1.05, 1.29)**		
Everyday Discrimination: Receive Poor Service at Restaurant or Store (Higher vs. Lower)		0.88 (0.76, 1.01)		
Everyday Discrimination: Treated as Not Smart (Higher vs. Lower)		**1.16 (1.02, 1.30)**		
Everyday Discrimination: People Act Afraid of You (Higher vs. Lower)		1.09 (0.94, 1.25)		
Everyday Discrimination: You are Threatened or Harassed (Higher vs. Lower)		1.17 (1.00, 1.35)		
Anticipated Discrimination (Mean Score)			**1.12 (1.07, 1.17)**	
Anticipated Discrimination: Prepare for Possible Insults Before Leaving Home (Higher vs. Lower)				1.01 (0.87, 1.14)
Anticipated Discrimination: Careful about Your Appearance in Order to Receive Good Service or Avoid Harassment (Higher vs. Lower)				1.08 (0.94, 1.23)
Anticipated Discrimination: Watch What You Say and How You Say It (Higher vs. Lower)				**1.15 (1.03, 1.27)**
Anticipated Discrimination: Avoid Certain Situations and Places (Higher vs. Lower)				1.13 (1.00, 1.25)

Note. Bold type indicates statistical significance as the 95% confidence interval (CI) includes the risk ratio and does not include 1. Regarding the structural variable, Model 1 includes Everyday Discrimination mean score; Model 2 includes Everyday Discrimination individual items; Model 3 includes Anticipated Discrimination mean score; and Model 4 includes Anticipated Discrimination individual items. Models 1–4 adjust for age, ethnicity, marital status, education, poverty status, urban–rural classification, insurance coverage source, deductible, difficulty paying medical bills, having a usual place for care, transportation barrier, and self-rated health.

**Table 7:** Logistic regression models of routine healthcare access within the past 12 months whether or not one had full prescription medications without delay (risk ratios (95%CI)).

Variable	Model 1	Model 2	Model 3	Model 4
LGB Women vs. Straight Men	**1.60 (1.16, 2.05)**	**1.56 (1.13, 1.99)**	**1.54 (1.11, 1.97)**	**1.51 (1.10, 1.93)**
Straight Women vs. Straight Men	**1.40 (1.20, 1.60)**	**1.38 (1.18, 1.58)**	**1.37 (1.17, 1.57)**	**1.37 (1.17, 1.57)**
LGB Men vs. Straight Men	**1.79 (1.21, 2.38)**	**1.77 (1.19, 2.34)**	**1.67 (1.10, 2.23)**	**1.67 (1.11, 2.23)**
Everyday Discrimination (Mean Score)	**1.36 (1.25, 1.46)**			
Everyday Discrimination: Treated with Less Courtesy or Respect (Higher vs. Lower)		1.18 (0.99, 1.37)		
Everyday Discrimination: Receive Poor Service at Restaurant or Store (Higher vs. Lower)		1.06 (0.87, 1.24)		
Everyday Discrimination: Treated as Not Smart (Higher vs. Lower)		**1.34 (1.12, 1.56)**		
Everyday Discrimination: People Act Afraid of You (Higher vs. Lower)		1.16 (0.92, 1.40)		
Everyday Discrimination: You are Threatened or Harassed (Higher vs. Lower)		1.23 (0.99, 1.47)		
Anticipated Discrimination (Mean Score)			**1.30 (1.22, 1.38)**	
Anticipated Discrimination: Prepare for Possible Insults Before Leaving Home (Higher vs. Lower)				**1.23 (1.01, 1.45)**
Anticipated Discrimination: Careful about Your Appearance in Order to Receive Good Service or Avoid Harassment (Higher vs. Lower)				1.18 (0.94, 1.42)
Anticipated Discrimination: Watch What You Say and How You Say It (Higher vs. Lower)				**1.24 (1.04, 1.44)**
Anticipated Discrimination: Avoid Certain Situations and Places (Higher vs. Lower)				**1.29 (1.08, 1.49)**

Note. Bold type indicates statistical significance as the 95% confidence interval (CI) includes the risk ratio and does not include 1. Regarding the structural variable, Model 1 includes Everyday Discrimination mean score; Model 2 includes Everyday Discrimination individual items; Model 3 includes Anticipated Discrimination mean score; and Model 4 includes Anticipated Discrimination individual items. Models 1–4 adjust for age, ethnicity, marital status, education, poverty status, urban–rural classification, insurance coverage source, deductible, difficulty paying medical bills, having a usual place for care, transportation barrier, and self-rated health.

**Table 8:** Mean differences in not utilizing a regular check-up within the last 2 years of LGB and straight people experiencing different levels of discrimination.

Discrimination	Straight	LGB	LGB-Straight w/Higher Level of Discrimination
Higher Level of Everyday Discrimination: Treated with Less Courtesy or Respect	0.19	0.31	***
Lower Level of Everyday Discrimination: Treated with Less Courtesy or Respect	0.17	0.18	-
Sig.	N.S.	***	-
Higher Level of Everyday Discrimination: Treated as Not Smart	0.21	0.33	**
Lower Level of Everyday Discrimination: Treated as Not Smart	0.20	0.20	-
Sig.	N.S.	***	-
Higher Level of Anticipated Discrimination: Watch What You Say and How You Say It	0.18	0.28	**
Lower Level of Anticipated Discrimination: Watch What You Say and How You Say It	0.19	0.19	-
Sig.	N.S.	*	-

Note. This table only includes statistically significant discrimination variables from the models. Regular Check-Up: 0 = Yes, 1 = No. Sig. = Significance. *** *p* < 0.001; ** *p* < 0.01; * *p* < 0.05; N.S. = Not Significant.

**Table 9:** Mean differences in not accessing full prescription medications or with delay within the past 12 months of LGB and straight people experiencing different levels of discrimination.

Discrimination	Straight	LGB	LGB-Straight w/Higher Level of Discrimination
Higher Level of Everyday Discrimination: Treated as Not Smart	0.22	0.31	*
Lower Level of Everyday Discrimination: Treated as Not Smart	0.07	0.15	-
Sig.	***	***	-
Higher Level of Anticipated Discrimination: Prepare for Possible Insults Before Leaving Home	0.16	0.29	**
Lower Level of Anticipated Discrimination: Prepare for Possible Insults Before Leaving Home	0.07	0.18	-
Sig.	**	**	-
Higher Level of Anticipated Discrimination: Watch What You Say and How You Say It	0.15	0.23	*
Lower Level of Anticipated Discrimination: Watch What You Say and How You Say It	0.09	0.15	-
Sig.	N.S.	*	-
Higher Level of Anticipated Discrimination: Avoid Certain Situations and Places	0.16	0.28	***
Lower Level of Anticipated Discrimination: Avoid Certain Situations and Places	0.10	0.12	-
Sig.	*	***	-

Note. This table only includes statistically significant discrimination variables from the models. Prescription Medication: 0 = Yes, 1 = No. Sig. = Significance. *** *p* < 0.001; ** *p* < 0.01; * *p* < 0.05; N.S. = Not Significant.

## Data Availability

National Center for Health Statistics. National Health Interview Survey. 2023. https://www.cdc.gov/nchs/nhis/documentation/2023-nhis.html (accessed on 3 December 2025).

## Supplementary Materials

The following supporting information can be downloaded at: https://www.mdpi.com/article/10.3390/ijerph22121830/s1, Table S1: Logistic Regression Models of Healthcare Utilization—Within the Last 2 Years Whether or Not One Had a Wellness Visit, Physical, or General Purpose Check-Up (Risk Ratios (95%CI)); Table S2: Logistic Regression Models of Healthcare Access—Within the Past 12 Months Whether or Not One Had Full Prescription Medications without Delay (Risk Ratios (95%CI)).
