# Supplementary material for: Examining the Intersectional and Structural Issues of Routine Healthcare Utilization and Access Inequities for LGB People with Chronic Diseases
Source: Int J Environ Res Public Health. 2025 Dec 6;22(12):1830. doi: 10.3390/ijerph22121830 (PMC12732992; doi:10.3390/ijerph22121830)
Supplement: Supplementary file 1 [file ijerph-22-01830-s001.zip › ijerph-3914605-supplementary.pdf]

Supplementary Materials

Table S1. Logistic Regression Models of Healthcare Utilization--Within the Last 2 Years Whether or Not One Had a Wellness Visit, Physical, or General Purpose Check-Up (Risk Ratios (95%CI)).

Variable	Model 1	Model 2	Model 3	Model 4
LGB Women vs. Straight Men	**1.29(1.02, 1.56)**	1.26(.99, 1.52)	**1.27(1.01, 1.54)**	**1.28(1.01, 1.55)**
Straight Women vs. Straight Men	.96(.87, 1.05)	.95(.86, 1.04)	.96(.87, 1.05)	.96(.87, 1.04)
LGB Men vs. Straight Men	1.01(.71, 1.32)	1.01(.71, 1.31)	.99(.69, 1.29)	.99(.69, 1.29)
Age (26-44 vs. 45-64)	.91(.82, .99)	.92(.83, 1.00)	.91(.83, 1.00)	.91(.83, 1.00)
Ethnicity (Hispanic vs. NH White)	.79(.68, .90)	.80(.69, .91)	.79(.68, .89)	.79(.68, .90)
Ethnicity (NH Black vs. NH White)	.60(.50, .70)	.61(.50, .72)	.60(.49, .70)	.60(.50, .71)
Ethnicity (NH Asian vs. NH White)	.78(.64, .93)	.79(.65, .94)	.78(.64, .93)	.79(.64, .94)
Marital Status (Not Married vs. Married)	.92(.83, 1.01)	.91(.83, 1.00)	.92(.84, 1.01)	.92(.84, 1.01)
Everyday Discrimination (Mean Score)	**1.15(1.08, 1.22)**			
Everyday Discrimination: Treated with Less Courtesy or Respect (Higher vs. Lower)		**1.17(1.05, 1.29)**		
Everyday Discrimination: Receive Poor Service at Restaurant or Store (Higher vs. Lower)		.88(.76, 1.01)		
Everyday Discrimination: Treated as Not Smart (Higher vs. Lower)		**1.16(1.02, 1.30)**		
Everyday Discrimination: People Act Afraid of You (Higher vs. Lower)		1.09(.94, 1.25)		
Everyday Discrimination: You are Threatened or Harassed (Higher vs. Lower)		1.17(1.00, 1.35)		
Anticipated Discrimination (Mean Score)			**1.12(1.07, 1.17)**	
Anticipated Discrimination: Prepare for Possible Insults before Leaving Home (Higher vs. Lower)				1.01(.87, 1.14)
Anticipated Discrimination: Careful about Your				1.08(.94, 1.23)

Appearance in Order to Get Good Service or Avoid Harassment (Higher vs. Lower)				
Anticipated Discrimination: Watch What You Say and How You Say It (Higher vs. Lower)				**1.15(1.03,1.27)**
Anticipated Discrimination: Avoid Certain Situations and Places (Higher vs. Lower)				1.13(1.00,1.25)
Education (Bachelor's Degree vs. Advanced Degree)	.93(.81, 1.05)	.94(.82, 1.05)	.93(.81, 1.05)	.93(.81, 1.05)
Education (Some College/Associate Degree vs. Advanced Degree)	.87(.75, .98)	.88(.76, .99)	.87(.76, .99)	.88(.76, 1.00)
Education (High School/Equivalent vs. Advanced Degree)	.80(.68, .92)	.81(.69, .93)	.81(.69, .93)	.82(.70, .94)
Education (Less than High School vs. Advanced Degree)	.81(.61, 1.00)	.82(.63, 1.02)	.81(.61, 1.00)	.82(.62, 1.02)
Poverty Status (In Poverty vs. Not in Poverty)	1.00(.80, 1.19)	.99(.80, 1.18)	.99(.80, 1.18)	1.00(.80, 1.19)
Urban-Rural Classification (Rural vs. Urban)	.95(.78, 1.11)	.94(.78, 1.11)	.95(.79, 1.11)	.95(.79, 1.11)
Insurance Coverage Source (Private Insurances: Others vs. Private Insurances: Employer/Union/Professional Organization)	.93(.78, 1.08)	.93(.78, 1.08)	.93(.78, 1.08)	.93(.78, 1.08)
Insurance Coverage Source (Public/Other Insurances vs. Private Insurances: Employer/Union/Professional Organization)	1.16(.93, 1.39)	1.17(.94, 1.40)	1.16(.93, 1.38)	1.16(.93, 1.39)
Deductible (Yes vs. No)	1.08(.93, 1.22)	1.08(.94, 1.23)	1.07(.93, 1.22)	1.08(.93, 1.22)
Deductible (Don't Know vs. No)	1.02(.75, 1.29)	1.02(.74, 1.29)	1.02(.75, 1.29)	1.02(.75, 1.29)
Difficulty Paying Medical Bills (Yes vs. No)	**1.41(1.22, 1.60)**	**1.40(1.21, 1.59)**	**1.41(1.22, 1.60)**	**1.42(1.23, 1.61)**
Having a Usual Place for Care (No vs. Yes)	**1.66(1.43, 1.90)**	**1.65(1.42, 1.89)**	**1.65(1.42, 1.89)**	**1.65(1.41, 1.88)**
Transportation Barrier (Yes vs. No)	1.08(.89, 1.26)	1.07(.89, 1.25)	1.07(.89, 1.25)	1.09(.90, 1.27)
Self-rated Health (Poor/Fair vs. Better Status)	**1.33(1.15, 1.51)**	**1.32(1.14, 1.50)**	**1.32(1.14, 1.50)**	**1.33(1.15, 1.51)**

Note. Bold type indicates statistical significance as the 95% confidence interval (CI) includes the risk ratio and does not include 1. Regarding the structural variable, Model 1 includes Everyday Discrimination mean score; Model 2 includes Everyday Discrimination individual items; Model 3 includes Anticipated Discrimination mean score; and Model 4 includes Anticipated Discrimination individual items.

Table S2. Logistic Regression Models of Healthcare Access--Within the Past 12 Months Whether or Not One Had Full Prescription Medications without Delay (Risk Ratios (95%CI)).

Variable	Model 1	Model 2	Model 3	Model 4
LGB Women vs. Straight Men	**1.60(1.16, 2.05)**	**1.56(1.13, 1.99)**	**1.54(1.11, 1.97)**	**1.51(1.10, 1.93)**
Straight Women vs. Straight Men	**1.40(1.20, 1.60)**	**1.38(1.18, 1.58)**	**1.37(1.17, 1.57)**	**1.37(1.17, 1.57)**
LGB Men vs. Straight Men	**1.79(1.21, 2.38)**	**1.77(1.19, 2.34)**	**1.67(1.10, 2.23)**	**1.67(1.11, 2.23)**
Age (26-44 vs. 45-64)	.84(.71, .96)	.84(.72, .96)	.85(.73, .97)	.86(.73, .98)
Ethnicity (Hispanic vs. NH White)	1.13(.91, 1.34)	1.12(.91, 1.34)	1.10(.88, 1.32)	1.11(.89, 1.34)
Ethnicity (NH Black vs. NH White)	1.13(.90, 1.35)	1.15(.92, 1.39)	1.12(.89, 1.35)	1.15(.91, 1.39)
Ethnicity (NH Asian vs. NH White)	.76(.45, 1.06)	.76(.46, 1.06)	.78(.48, 1.09)	.79(.48, 1.10)
Marital Status (Not Married vs. Married)	**1.20(1.02, 1.38)**	**1.20(1.02, 1.38)**	**1.21(1.03, 1.39)**	**1.21(1.03, 1.40)**
Everyday Discrimination (Mean Score)	**1.36(1.25, 1.46)**			
Everyday Discrimination: Treated with Less Courtesy or Respect (Higher vs. Lower)		1.18(.99, 1.37)		
Everyday Discrimination: Receive Poor Service at Restaurant or Store (Higher vs. Lower)		1.06(.87, 1.24)		
Everyday Discrimination: Treated as Not Smart (Higher vs. Lower)		**1.34(1.12, 1.56)**		
Everyday Discrimination: People Act Afraid of You (Higher vs. Lower)		1.16(.92, 1.40)		
Everyday Discrimination: You are Threatened or Harassed (Higher vs. Lower)		1.23(.99, 1.47)		
Anticipated Discrimination (Mean Score)			**1.30(1.22, 1.38)**	
Anticipated Discrimination: Prepare for Possible Insults before Leaving Home (Higher vs. Lower)				**1.23(1.01, 1.45)**
Anticipated Discrimination: Careful about Your Appearance in Order to Get Good Service or Avoid Harassment (Higher				1.18(.94, 1.42)

vs. Lower)				
Anticipated Discrimination: Watch What You Say and How You Say It (Higher vs. Lower)				**1.24(1.04, 1.44)**
Anticipated Discrimination: Avoid Certain Situations and Places (Higher vs. Lower)				**1.29(1.08, 1.49)**
Education (Bachelor's Degree vs. Advanced Degree)	.97(.75, 1.18)	.96(.75, 1.18)	.96(.75, 1.16)	.96(.75, 1.17)
Education (Some College/Associate Degree vs. Advanced Degree)	1.25(.98, 1.51)	1.26(.99, 1.53)	1.26(.99, 1.53)	1.27(1.00, 1.55)
Education (High School/Equivalent vs. Advanced Degree)	1.19(.91, 1.47)	1.19(.91, 1.48)	1.22(.93, 1.50)	1.21(.93, 1.50)
Education (Less than High School vs. Advanced Degree)	1.10(.71, 1.49)	1.11(.71, 1.50)	1.09(.70, 1.48)	1.10(.71, 1.50)
Poverty Status (In Poverty vs. Not in Poverty)	.99(.73, 1.25)	.98(.73, 1.24)	.96(.70, 1.21)	.97(.71, 1.22)
Urban-Rural Classification (Rural vs. Urban)	1.01(.81, 1.21)	1.01(.80, 1.21)	1.02(.82, 1.22)	1.03(.82, 1.23)
Insurance Coverage Source (Private Insurances: Others vs. Private Insurances: Employer/Union/Professional Organization)	**1.54(1.25, 1.84)**	**1.54(1.25, 1.84)**	**1.53(1.24, 1.82)**	**1.54(1.24, 1.83)**
Insurance Coverage Source (Public/Other Insurances vs. Private Insurances: Employer/Union/Professional Organization)	.89(.65, 1.12)	.90(.66, 1.14)	.88(.64, 1.12)	.89(.65, 1.13)
Deductible (Yes vs. No)	1.23(.97, 1.50)	1.24(.97, 1.51)	1.22(.95, 1.48)	1.22(.96, 1.49)
Deductible (Don't Know vs. No)	**1.59(1.08, 2.09)**	**1.57(1.07, 2.07)**	**1.59(1.09, 2.09)**	**1.60(1.09, 2.10)**
Difficulty Paying Medical Bills (Yes vs. No)	**3.19(2.71, 3.68)**	**3.21(2.72, 3.69)**	**3.22(2.72, 3.72)**	**3.26(2.76, 3.77)**
Having a Usual Place for Care (No vs. Yes)	1.36(1.00, 1.71)	1.35(.99, 1.70)	1.33(.98, 1.69)	1.33(.98, 1.68)
Transportation Barrier (Yes vs. No)	**1.70(1.33, 2.08)**	**1.71(1.34, 2.08)**	**1.69(1.32, 2.07)**	**1.73(1.35, 2.11)**
Self-rated Health (Poor/Fair vs. Better Status)	**1.63(1.35, 1.90)**	**1.62(1.35, 1.90)**	**1.60(1.33, 1.87)**	**1.62(1.34, 1.89)**

Note. Bold type indicates statistical significance as the 95% confidence interval (CI) includes the risk ratio and does not include 1. Regarding the structural variable, Model 1 includes Everyday Discrimination mean score; Model 2 includes Everyday Discrimination individual items; Model 3 includes Anticipated Discrimination mean score; and Model 4 includes Anticipated Discrimination individual items.
